# Streptococcal group B integrative and mobilizable element IMESag-*rpsI* encodes a functional relaxase involved in its transfer

**DOI:** 10.1098/rsob.160084

**Published:** 2016-10-05

**Authors:** Fabian Lorenzo-Diaz, Cris Fernández-Lopez, Pierre-Emmanuel Douarre, Adrian Baez-Ortega, Carlos Flores, Philippe Glaser, Manuel Espinosa

**Affiliations:** 1 Departamento de Bioquímica, Microbiología, Biología Celular y Genética, Instituto Universitario de Enfermedades Tropicales y Salud Pública de Canarias, Universidad de La Laguna, Av. Astrofísico Francisco Sánchez s/n, 38071 Santa Cruz de Tenerife, Spain; 2 Unidad de Investigación, Hospital Universitario Nuestra Señora de Candelaria, Santa Cruz de Tenerife, Spain; 3 Centro de Investigaciones Biológicas, CSIC, Madrid, Spain; 4 Institut Pasteur, Unité Ecologie et Evolution de la Résistance aux Antibiotiques, Paris CNRS UMR3525, France; 5 Department of Veterinary Medicine, University of Cambridge, Madingley Road, Cambridge, UK; 6 CIBER de Enfermedades Respiratorias, Instituto de Salud Carlos III, Madrid, Spain

**Keywords:** conjugation, horizontal gene transfer, integrative and mobilizable elements mobilization, relaxases, *Streptococcus*

## Abstract

*Streptococcus agalactiae* or Group B *Streptococcus* (GBS) are opportunistic bacteria that can cause lethal sepsis in children and immuno-compromised patients. Their genome is a reservoir of mobile genetic elements that can be horizontally transferred. Among them, integrative and conjugative elements (ICEs) and the smaller integrative and mobilizable elements (IMEs) primarily reside in the bacterial chromosome, yet have the ability to be transferred between cells by conjugation. ICEs and IMEs are therefore a source of genetic variability that participates in the spread of antibiotic resistance. Although IMEs seem to be the most prevalent class of elements transferable by conjugation, they are poorly known. Here, we have studied a GBS-IME, termed IMESag-*rpsI*, which is widely distributed in GBS despite not carrying any apparent virulence trait. Analyses of 240 whole genomes showed that IMESag-*rpsI* is present in approximately 47% of the genomes, has a roughly constant size (approx. 9 kb) and is always integrated at a single location, the 3′-end of the gene encoding the ribosomal protein S9 (*rpsI*). Based on their genetic variation, several IMESag-*rpsI* types were defined (A–J) and classified in clonal complexes (CCs). CC1 was the most populated by IMESag-*rpsI* (more than 95%), mostly of type-A (71%). One CC1 strain (*S. agalactiae* HRC) was deep-sequenced to understand the rationale underlying type-A IMESag-*rpsI* enrichment in GBS. Thirteen open reading frames were identified, one of them encoding a protein (MobSag) belonging to the broadly distributed family of relaxases MOB_V1_. Protein MobSag was purified and, by a newly developed method, shown to cleave DNA at a specific dinucleotide. The *S. agalactiae* HRC-IMESag-*rpsI* is able to excise from the chromosome, as shown by the presence of circular intermediates, and it harbours a fully functional mobilization module. Further, the *mobSag* gene encoded by this mobile element is able to promote plasmid transfer among pneumococcal strains, suggesting that MobSag facilitates the spread of IMESag-*rpsI* and that this spread would explain the presence of the same IMESag-*rpsI* type in GBS strains belonging to different CCs.

## 1. Introduction

Conjugation is the major process involved in horizontal gene transfer (HGT) and is responsible for the spreading of antibiotic resistance and virulence traits among bacterial populations. Therefore, conjugation is a primary driving force that operates on evolution of bacteria and on their colonization of different niches [1,2]. Transfer of a conjugative genetic element is initiated by an element-encoded protein, termed relaxase. Relaxases are site-specific endonucleases that cleave the DNA to be transferred at a specific region, the origin of transfer (*oriT*). Cleavage is exerted at the phosphodiester bond of a specific dinucleotide in one of the DNA strands. The relaxase-mediated nucleophilic attack generates a stable DNA–protein complex that blocks the 5′-end and leaves a free 3′-OH end in the DNA to be transferred (t-DNA). The t-DNA : relaxase complex is actively pumped to the recipient cell by the coupling protein and the type IV secretion system (T4SS). Finally, the t-DNA in the recipient cell replicates, most probably, through lagging-strand synthesis [3].

An excellent example of the influence of conjugation on the bacterial lifestyle is represented by the Gram-positive pathogenic bacterium *Streptococcus agalactiae*, or Group B *Streptococcus* (GBS), within the phylum Firmicutes. This opportunistic bacterium causes nosocomial infections in newborns and is responsible for invasive diseases in immuno-compromised adults and elderly populations, causing sepsis in some cases [4]. The chromosome of *S. agalactiae* shows a complex organization and arrangements, where the presence of a conserved backbone and up to 69 variable regions have been observed [5,6]. A comparative analysis of the first eight sequenced genomes of GBS showed that two-thirds of the variable regions correspond to putative integrative and conjugative elements (ICEs) and related elements, such as integrative and mobilizable elements (IMEs). This analysis permitted the identification of 12 ICEs and six IMEs that were placed in 15 different locations in the chromosome of the bacterium [7]. These elements behave as transmissible DNA regions that can excise from the donor chromosome to form a circular molecule that can be either self-transferred (ICE) or mobilized by an auxiliary element (IME) to a recipient cell [8–10]. After conjugative transfer (with or without replication), the element is inserted into the chromosome of the recipient host. These mobile elements are considered as the most abundant source of HGT in prokaryotes [11–13] and, therefore, they could be key factors to explain the high plasticity and complexity found in GBS genomes.

A recent report suggested that IMEs are more frequent than ICEs [14]. However, studies that focused on the prevalence, diversity and functionality of IMEs are still scarce. One remarkable example of IMEs found in the GBS is IMESag-*rpsI*, which is integrated at the 3′-end of the gene encoding the ribosomal protein S9 (*rpsI*) and is one of the six IME families in GBS [11]. IMESag-*rpsI* was first found in six out of eight sequenced GBS strains [7]. Curiously enough, this IME does not carry an obvious cargo gene that could provide a selective advantage that could explain its ubiquity. To understand how a frequent IME with no apparent selective advantage is maintained in the GBS population, here we studied the mobilization mechanisms of IMESag-*rpsI* as a case study of IME in Firmicutes, whose presence could not be explained simply by selection. We chose a previously uncharacterized human clinical isolate *S. agalactiae*, sequenced its entire genome and performed a complete follow-up of the encoded IMESag-*rpsI*. Comparative analysis of the distribution and variability of the IMESag-*rpsI* in 240 GBS genomes belonging to different multilocus sequence types (MLST) provided us with clues on the mobility of this element. *In silico* analyses indicated the existence of a gene encoding a putative relaxase (termed MobSag) with a HUH motif, which constitutes the signature of the relaxases [15]. Functional studies with purified MobSag protein showed that it was able to cleave supercoiled plasmid DNAs harbouring its target (the *oriT* of IMESag-*rpsI*) *in vitro*; unequivocal determination of the nick site was achieved by a novel and simple method developed in this work. In addition, IMESag-*rpsI* was able to mediate the intra-species conjugative mobilization of the IMESag-*rpsI in vivo*. The combination of *in silico* and functional analyses of IMESag-*rpsI* provided a comprehensive view of the vertical and horizontal transfer history of this element.

## 2. Material and methods

### 2.1. Bacterial strains, plasmids and oligonucleotides

The bacterial strains and plasmids used in this work are listed in table 1. *Streptococcus agalactiae* HRC, a serotype V representative strain belonging to the tetracycline-resistance CC1 lineage Tn*916*-1 [18], was isolated in 2009 from a 60-year-old woman with abdominal sepsis at the Hospital Ramón y Cajal (Madrid, Spain). *Streptococcus pneumoniae* cells were grown in AGCH media as reported [24,25]. *Streptococcus agalactiae* and *E. coli* cells were grown in tryptone-yeast (TY) media (Pronadisa, Spain). Antibiotics were used at the following concentrations: 10 µg ml^−1^ novobiocin, 1 µg ml^−1^ tetracycline, and 1 µg ml^−1^ erythromycin for *S. pneumoniae*; in the case of *S. agalactiae*, 4 µg ml^−1^ tetracycline, 4 µg ml^−1^ erythromycin, 50 µg ml^−1^ rifampicin, and 10 µg ml^−1^ fusidic acid; for *E. coli*, 50 µg ml^−1^ kanamycin. All strains were grown at 37°C, with (*E. coli*) or without (*S. agalactiae* and *S. pneumoniae*) aeration. Purification of DNA from plasmids pMV158, pMVS and pMVSag was performed by two consecutive CsCl gradients as previously described [26]. Plasmids pET24b and pMobSag were purified with the high pure plasmid isolation kit (Roche Applied Science, Indianapolis, IN). Oligonucleotides used are listed in the electronic supplementary material, table S1.

**Table 1.** Bacterial strains and plasmids.

bacterial strains	description or genotype^a^	source or reference
*Streptococcus agalactiae*		
HRC	human isolate	this work
NEM316	human isolate	[16]
BM132	human isolate; Rif^R^, Fus^R^	[17]
Spain-IP-33	cow isolate	[5]
H36B	human isolate	[5]
CCH208800879	human isolate	[18]
2603V/R	human isolate	[19]
18RS21	human isolate	[5]
Spain-54	human isolate	[18]
Madagascar-IP-7	human isolate	[18]
COH1	human isolate	[5]
*Streptococcus pneumoniae*		
708	*end*-1, *exo*-1, *trt*-1, *hex*-4, *malM594*	[20]
MP3008	*end*-1, *exo*-1, *trt*-1, *hex*-4; Nov^R^	[3]
*Escherichia coli*		
BL21 (DE3)	λ DE3 (*lacI lac*UV5-T7 gene 1 *ind*1 *sam*7 *nin*5) F^−^ *dcm ompT hsdS* (*r*_B_*^−^m*_B_*^+^*) *gal*	[21]
plasmids (bp)	description^a^	
pMV158 (5.540)	originally isolated from *S. agalactiae* MV158; Tc^R^	[22]
pMVS (3.687)	pMV158-derivative lacking *oriT*_pMV158_, *mobM* and *ssoA*_pMV158_; insertion of *Stu*I restriction site; Tc^R^	this work
pMVSag (5.289)	pMVS derivative with mobilization module (*oriT*_Sag_*, mobSag*, *ssoA*_Sag_) from *S. agalactiae* HRC chromosome; Tc^R^	this work
pET24b (5.309)	expression vector under control of ϕ10 promoter from T7 phage; Km^R^	Novagen
pMobSag (6.598)	pET24b containing *mobSag* gene; Km^R^	this work
pAMβ1 (27.815)	conjugative plasmid from *E. faecalis* DS5; Em^R^	[23]

^a^Rif^R^, Fus^R^, Nov^R^, Tc^R^, Km^R^ and Em^R^: resistance to rifampicin, fusidic acid, novobiocin, tetracycline, kanamycin and erythromicin, respectively.

### 2.2. Bioinformatic analysis

A collection of 240 GBS genomes was used for phylogenetic analysis: 229 of them were reported previously by Da Cunha *et al*. [18], an other 10 were available at GenBank (SS1, NZ_CP010867.1; 09mas018883, NC_021485.1; NGBS061, NZ_CP007631.1; A909, NC_007432.1; GD201008-001, NC_018646.1; 2603V/R, NC_004368.1; GBS2-NM, NZ_CP007571.1; GBS6, NZ_CP007572.1; GBS1-NY, NZ_CP007570.1; NEM316, NC_004368.1), and another one was sequenced in this work (HRC, see below). MobM-related relaxases were searched in whole genomes by using the 300 N-terminal amino acid residues of the MobM relaxase of plasmid pMV158; they were used as the query in a PSI-BLAST search (*e*-value: 1 × 10^−6^, limited to 100 non-redundant hits), and the homologues found were aligned using the program MUSCLE as described previously for the family of plasmid-encoded relaxases related to MobM [27]. The core genome phylogeny of these 240 GBS strains was performed using the parsnp program from the Harvest suite [28]. The core genome SNPs of all strains were aligned against the reference genome sequence of the 2603 V/R strain (NC_004116) using MUSCLE and ignoring MUMi recruitment regions of recombination; alignments were filtered using PhiPack [29] and the phylogeny was reconstructed by FastTree2 [30]. The tree was annotated using iTOL: interactive tree of life [31], an online phylogenetic tree viewer (http://itol.embl.de/). The limits of the mobile elements (prophages, ICEs, IMEs, CIMEs) were determined after identification of the attachment (*att*) sites using the Repeat Finder program (http://www.cbcb.umd.edu/software/RepeatFinder/). Blastn and Easy fig 2.2.2 [32] were used for comparative sequence analysis of the identified IMEs, classified in groups (types) based on a threshold difference of more than five SNPs in the whole element (IMESag-*rpsI* DNA sequences will be provided upon request). In order to evidence if recombination was involved in contributing to diversity of IMESag-*rpsI*, we used serially SBP and GARD algorithms [33] available as online tools (http://www.datamonkey.org/dataupload.php). We used the HKY85 nucleotide substitution model in both cases. SBP was used to detect the presence of recombination. GARD was then used to identify the number of breakpoints and their approximate positions. Consistency of results was pursued by running GARD considering constant rates of diversity across sites, or using a beta-gamma distribution of rates across sites.

### 2.3. Whole genome sequencing

The *S. agalactiae* HRC genome was sequenced at the Institute of Tropical Diseases and Public Health of the Canary Islands (University of La Laguna, Tenerife, Spain). The library was constructed in the AB Library Builder system (Thermo Fisher Scientific, Inc.) and its length, size distribution and concentration were assessed in the Bioanalyzer 2100 (Agilent Technologies, Santa Clara, CA). Samples were subjected to emulsion PCR and enrichment by the OneTouch system (using the Ion OneTouch 200 Template v2DL kit), loaded in one Ion314 chip and sequenced in the Ion Torrent Personal Genome Machine (PGM) platform (Thermo Fischer Scientific, Inc.) with the 200 bp sequencing chemistry. A total of 121 Mb was obtained (686 800 reads, mean read length approximately 177 bp). Raw sequence data were deposited in the Sequence Read Archive under the accession number SRP060555. The IonGAP web platform (http://iongap.hpc.iter.es) was used for performing genome assembly, gene prediction, SNP calling and functional annotation. The *S. agalactiae* strain SS1 genome sequence (CP010867.1) was employed as reference sequence for SNP calling. A total of 35 contigs (more than 500 bp) were assembled, containing a total of 2 198 489 bp (mean coverage depth 50×), with an N50 length of 173 378 bp. Loci previously described as hotspot insertion sites [6] were analysed in the HRC genome for the presence of ICEs and related elements. Comparison between the available genome sequences of *S. agalactiae* at NCBI and the genome of HRC led to the characterization of the identified elements and the detection of new insertion sites.

### 2.4. DNA cloning

**Figure 1.**
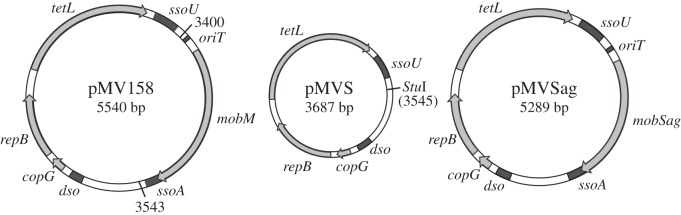
Genetic maps of plasmids pMV158, pMVS and pMVSag. Plasmid regions containing the origins of leading- (*dso*) and lagging-strand (*ssoU* and *ssoA*) synthesis, and the origin of transfer (*oriT*) are depicted in black. Genes are represented by grey arrows (arrowheads, direction of synthesis): *copG* and *repB*, involved in plasmid replication and its control; *tetL*, tetracycline-resistance determinant of type L; and *mobM* and *mobSag*, encoding conjugative mobilization proteins. Black lines (coordinates 3400–3543 of pMV158) encompass the region of pMV158 (NC_010096.1) that was deleted to construct its derivative pMVS. The single *Stu*I restriction site generated in pMVS (coordinate 3545 of pMV158) is also illustrated. The IMESag-*rpsI* region including the *oriT*, *mobSag* gene and *ssoA* (1.602 bp) was amplified from *S. agalactiae* HRC and cloned into the *Stu*I site of pMVS to obtain plasmid pMVSag.

Genomic DNA was extracted using the Qiagen DNeasy kit (Qiagen, Hilden, Germany). The recombinant plasmid pMVS was constructed by inverse PCR, using the INV-F and INV-R phosphorylated oligonucleotides (listed in the electronic supplementary material, table S1) and DNA of plasmid pMV158 as template, followed by auto-ligation and transformation into the *S. pneumoniae* 708 strain as previously described [34]. The fragment from IMESag-*rpsI* including the *oriT*_Sag_, *mobSag* gene and *ssoA*_Sag_ was amplified by PCR, using primers Sag2 (electronic supplementary material, table S1) and genomic DNA from *S. agalactiae* HRC. This fragment was cloned into the single *Stu*I site of pMVS and recovered by transformation of *S. pneumoniae* 708 to obtain plasmid pMVSag. The same fragment was used for a nested-PCR with Sag1 primers (electronic supplementary material, table S1), and the product obtained was then digested by *Nhe*I and *Xho*I enzymes and cloned into pET24b to construct the pMobSag plasmid. Genetic maps of plasmids pMV158, pMVS and pMVSag are depicted in figure 1. PCR reactions were done with the DNA polymerase Phusion enzyme (Thermo Fisher Scientific, Inc.) following the manufacturer's instructions. Constructions were confirmed by Sanger DNA sequencing (Secugen S.L., Spain).

### 2.5. Protein purification and sequencing

*E. coli* BL21 (DE3) cells were transformed [35] with pMobSag DNA and used to overproduce protein MobSag. Protein purification was performed as reported [36]. The concentration of MobSag was measured with a Nanodrop ND-1000 spectrophotometer, and the N-terminal sequence of the protein was analysed in a Procise 494 protein sequencer (Perkin Elmer, Foster City, CA) by CIB-CSIC (Madrid, Spain).

### 2.6. Functional assays and nick site determination

Relaxation of supercoiled plasmid DNA by purified MobSag protein was performed as previously described [37]. Reactions were carried out by incubating supercoiled DNA of plasmids pMVS or pMVSag (8 nM) without or with protein MobSag in buffer B (20 mM Tris-HCl pH 7.6, 0.1 mM EDTA, 1% glycerol (w/v), 1 mM dithiothreitol, and 8 mM MnCl_2_) for 20 min. Different protein concentrations (from 8 to 418 nM), incubation temperatures (from 20 to 70°C) and times (from 1 to 40 min) were tested. Generation of relaxed (FII forms) from supercoiled DNA (FI forms) was analysed by electrophoresis on 1% agarose gels stained with GelRed 1×. Densitometric analyses were done using the QuantityOne software (Bio-Rad USA, Hercules, CA). The amount of relaxed DNA forms observed in the control was subtracted in each assay. To determine the phosphodiester bond cleaved by MobSag, the FII plasmid forms were purified from the gel with the QIAquick Gel Extraction Kit (Qiagen, Hilden, Germany), and then subjected to Sanger DNA sequencing (Secugen S.L., Madrid, Spain), using the primer nickSag-R (electronic supplementary material, table S1).

### 2.7. Filter-mating conjugal transfer assays

Mobilization assays of pMV158, pMVS and pMVSag from *S. pneumoniae* 708 donor colony-forming units (CFU) harbouring pAMβ1 (as the auxiliary plasmid) and *S. pneumoniae* MP3008 (resistant to novobiocin) as recipients were performed as previously described [3,38]. Donor and recipient cultures were grown at 37°C to 5 × 10^8^ cells ml^−1^, mixed at a ratio of 1 (donor) to 2 (recipient), and passed through sterile nitrocellulose filters (0.22 μm pore size; Millipore, Bedford, MA). The filters were placed upside down on another filter, and placed on a plate with conjugation medium (AGCH with 10 mM MgCl_2_, 0.2% albumin, 0.2% glucose, 2% agar and 5 µg ml^−1^ DNase I). After 4 h of incubation at 37°C, the cells were recovered and transconjugants and recipients were selected by plating on AGCH supplemented with the appropriate antibiotics. Five independent experiments were performed, and mobilization efficiencies were calculated as the number of transconjugants per recipient CFU.

## 3. Results

### 3.1. Distribution and diversity of IMESag-*rpsI* in GBS

**Figure 2.**
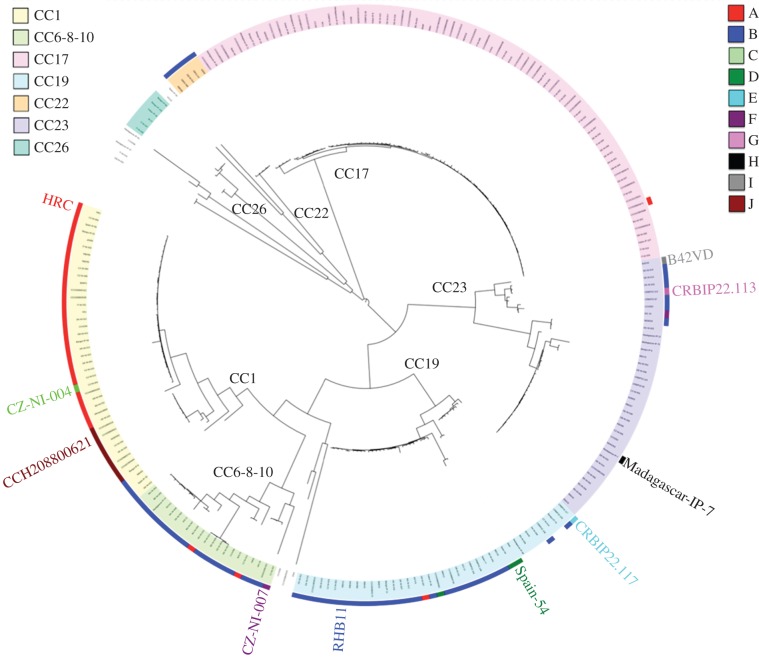
Core genome phylogeny and distribution of the IMESag-*rpsI* types within different GBS clonal complexes (CCs). The core genome of 240 GBS strains was obtained as indicated in Material and methods, and compared to the *S. agalactiae* 2603V/R reference genome (NC_004116). The iTOL viewer (http://itol.embl.de/) was used to annotate the phylogenetic tree. The seven different clonal complexes are represented with coloured leaves and the 10 different IMESag-*rpsI* types are highlighted with colour strips. One reference strain carrying each IMESag-*rpsI* type is denoted on the tree (see electronic supplementary material, table S2). One thousand bootstraps were employed in order to obtain the confidence values for internal branches, and bootstrap values more than 50% were used for representing the consensus phylogeny.

**Figure 3.**
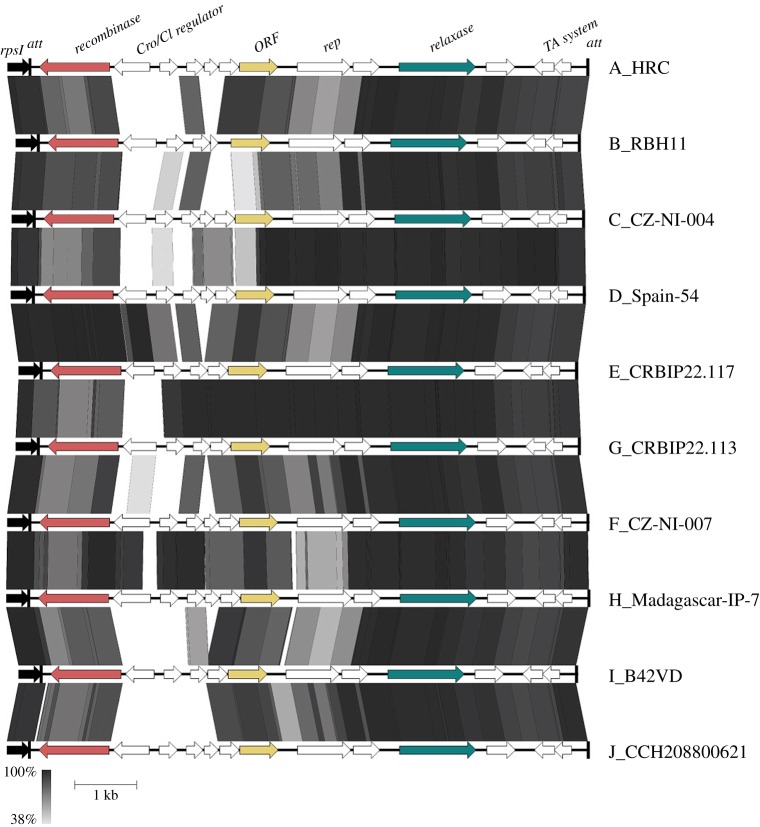
Open reading frame (ORF) organization and comparison of the 10 different IMESag-*rpsI* types (A to J) found in the analysed genomes. The representative strain of each type is indicated at the right of the figure (see electronic supplementary material, table S2). In the upper part, the names of identified ORFs are shown. The two putative recombination breakpoints found by GARD [36] were located inside a gene encoding a conserved hypothetical protein (coloured in ochre). The sequence identity percentage is shown in grey scale and ranges between 100 and 38%.

We performed a detailed phylogenetic characterization of 240 GBS isolates and found that IMESag-*rpsI* [7] carries an open reading frame that could encode a relaxase belonging to the MOB_V1_ family. The prototype of this family is MobM, encoded by the promiscuous plasmid pMV158, which, interestingly, was primarily identified in a clinical isolate of *S. agalactiae* [22]. MOB_V1_ relaxases are overrepresented in mobilizable elements from Firmicutes, a clade including genera of outstanding relevance in the spread of antibiotic resistance traits, such as *Staphylococcus*, *Enterococcus* or *Streptococcus* [27]. We thus reasoned that a global study of the distribution and the diversity of IMESag-*rpsI*, mediated by the MOB_V1_ relaxase it encodes, could help us to understand the dissemination of this particular IME. IMESag-*rpsI* was found in 112 out of the 240 strains analysed, showing a nearly uniform size between 9.1 and 9.5 kb. The element was found to be widely distributed in clonal complexes (CC) CC1, CC6-8-10, CC19 and CC22 (figure 2 and table 2), but was virtually absent in the hyper-virulent CC17 strains, as well as in the ST23 serotype Ia lineage that includes human isolates. We did not find any strain carrying two or more IMESag-*rpsI*. The genetic organization of IMESag-*rpsI* was highly conserved among all analysed strains, although sequence alignment of the 112 IMESag-*rpsI* detected allowed us to distinguish between 10 different types based on nucleotide divergence (named from A to J; figure 3; electronic supplementary material, table S2). The highest variability was found in the region encoding the transcriptional regulator Cro/CI and the three open reading frames (ORFs) upstream (59.8–99.9% divergence; figure 3). The analyses of recombination in DNA sequences of the 10 different types provided strong support for the existence of two breakpoints 127 bp away from each other within a gene predicted to encode a conserved hypothetical protein (NCBI ref. seq. WP_000591148; figure 3). Analysis of the distribution of the different types according to the phylogeny (figure 2 and table 2) suggested not only vertical but also horizontal transmission of the different IMESag-*rpsI* variants. The presence of the same IMESag-*rpsI* type in strains belonging to different CCs can be explained by (i) single acquisition (horizontal transfer) followed by clonal dissemination (vertical transmission), as for instance, type-A in CC1, and (ii) replacement of a resident element by another acquired by horizontal transfer (for instance, substitution of type-A for a type-C element in strain CZ-NI-004).

**Table 2.** IMESag-*rpsI* distribution across different GBS clonal complexes.

IMESag-*rpsI* type^a^	no isolates								total
	CC1	CC6-8-10	CC17	CC19	CC22	CC23	CC26	others	
A (HRC)	29	2	1	1					33
B (RBH11)	3	18		29	5	6			61
C (CZ-NI-004)	1								1
D (Spain-54)				3					3
E (CRBIP22.117)				1					1
F (CZ-NI-007)		1				1			2
G (CRBIP22.113)						1			1
H (Madagascar-IP-7)						1			1
I (B42VD)						1			1
J (CCH208800621)	8								8
with IMESag-*rpsI*	41	21	1	34	5	10	0	0	112
total number of isolates	43	21	80	40	5	37	6	8	240

^a^The representative strain of each IMESag-*rpsI* type is indicated in parentheses. Criteria for defining IMESag-*rpsI* types are explained in Material and methods (see figure 2 and electronic supplementary material, table S2, for further details).

### 3.2. Genetic structure of the type-A IMESag-*rpsI*

**Figure 4.**
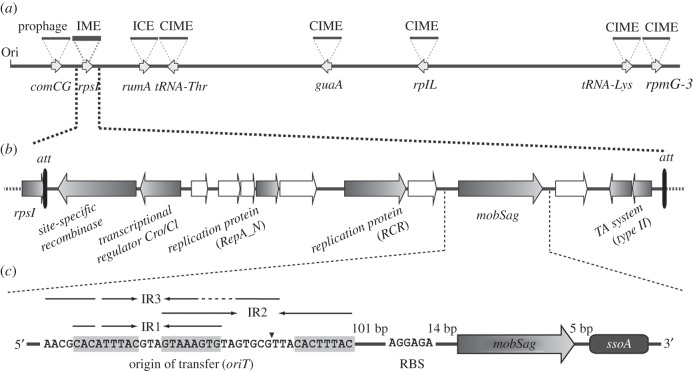
Genetic structure of the IMESag-*rpsI* of *S. agalactiae* HRC strain. (*a*) Schematic map of the *S. agalactiae* HRC chromosome indicating the relative positions of putative ICEs, CIMEs and a prophage upstream of the IMESag-*rpsI* (thicker lines). The genes in which the elements are integrated are indicated beneath. The origin of replication of the chromosome (Ori) is also shown. All elements matched with homologous elements found in the *S. agalactiae* sequenced genomes except for the ICE inserted in the *rumA* gene, which belongs to the ICE*Sp2905*-like family of elements found in *S. pyogenes* [42]. (*b*) Genetic map of the IME integrated 3′ to the *rpsI* gene (IMESag-*rpsI*; 9496 bp). Open reading frames (ORFs) with annotated functions are depicted by grey-shadowed arrows, whereas ORFs with unknown function are depicted in white. Attachment sites (*att*) are indicated with black lines. (*c*) Detailed map of the IMESag-*rpsI* mobilization module (1555 bp). From 5′ to 3′ the following elements are depicted: origin of transfer (*oriT*), ribosome-binding site sequence (RBS), *mobSag* gene (1257 bp) and the putative lagging-strand replication origin (*ssoA*). Three inverted repeats within the *oriT* (IR1, IR2 and IR3) are indicated by inverted arrows with the core sequence shadowed in grey. The predicted phosphodiester bond including the nick site (G/T) is depicted by an arrowhead inside the *oriT* sequence. Nucleotide distances between the represented elements are indicated in base pairs (bp).

Vertical and horizontal transfer processes may explain the presence of the same IMESag-*rpsI* type in strains belonging to different CCs. In addition, our analyses on the distribution of this IME showed that CC1 was the most populated by IMESag-*rpsI* (more than 95% of the members), mainly belonging to type-A (figure 2 and table 2). Thus, and as a representative of these CC1 strains, we decided to sequence the genome of *S. agalactiae* HRC and perform an in-depth analysis by using the IonGAP platform [39]. The assembled contigs were compared to the different available *S. agalactiae* genomes (electronic supplementary material, table S3), and the SS1 human strain (NZ_CP010867.1) was found to be the closest reference belonging to Sequence Type (ST) 1. Analysis of the mobilome of *S. agalactiae* HRC allowed us to determine that it carries the plasmid pST2426, previously identified in *Streptococcus tigurinus* [40,41], as well as eight putative ICEs and related mobile genetic elements (figure 4*a*; electronic supplementary material, table S4). The type-A IMESag-*rpsI* encoded by *S. agalactiae* HRC spans 9496 bp and is flanked by two attachment sites (*att*). This IME contains 13 ORFs, eight of them annotated as: a site-specific recombinase (Phage_integrase; PF00589), a transcriptional regulator from the Cro/CI family (HTH_3; PF01381), the N-terminus of an initiator protein A (RepA_N; PF06970), a replication initiator belonging to the rolling circle family (Rep_trans; PF02486), a conjugative relaxase from the MOB_V_ family (Mob_Pre; PF01076), and a type II toxin–antitoxin system from the RelBE family (RelB; PF04221 and RelE; PF06296; figure 4*b*).

### 3.3. Generation of IMESag-*rpsI* extrachromosomal intermediates

**Figure 5.**
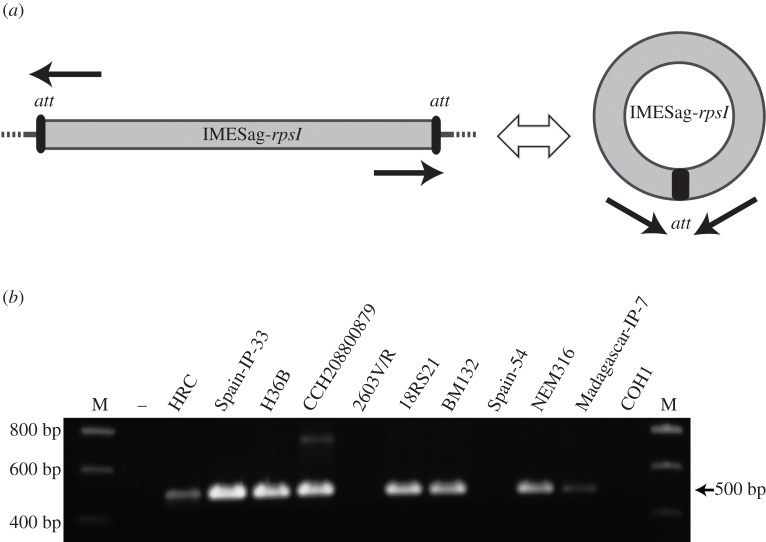
Identification of extrachromosomal circular forms of IMESag-*rpsI* in different *S. agalactiae* strains. (*a*) Schematic representation of the excision as a circular molecule from the chromosomal IMESag-*rpsI*. Primers used to detect excised forms are illustrated as black arrows. (*b)* Electrophoretic analysis of the PCR product (494 bp) to detect circular forms of IMESag-*rpsI*. Genomic DNA (500 ng) from selected strains (indicated above the image; table 2) was used in PCR reactions with oligonucletides cIME-F and cIME-R (electronic supplementary material, table S1). Positive (NEM316) and negative (COH1) control strains are included, and the primers used in both cases had identical sequences. Molecular sizes (in basepairs) of the DNA ladder (M, DNA SmartLadder MW-1700-10) are indicated on the left.

Excision from the chromosome and subsequent circularization of an IME is the first step of its transfer lifecycle. To determine whether IMESag-*rpsI* generated extrachromosomal molecules, we designed a PCR strategy employing divergent primers that detect the presence of circular forms of IMESag-*rpsI* (figure 5*a*). We selected 11 phylogenetically divergent GBS isolates to perform the same PCR analysis (figure 5*b*). The ST17 strain COH1 (HG939456) that lacks IMESag-*rpsI*, and the ST23 strain NEM316 (AL732656) whose IMESag-*rpsI* was previously identified as a circular molecule [6] were used as negative and positive controls, respectively. The results showed that seven out of nine strains (excluding controls) generated circular excised forms of IMESag-*rpsI*. In the two remaining strains, 2603V/R and Spain-54, the IME does not excise or it does so at a very low rate, undetectable under the experimental conditions applied. Interestingly, these two strains are closely related; both of them are CC19 serotype V strains and share the same type-D IMESag-*rpsI*. In this IME, the putative transcriptional regulator Cro/CI is less than 30% identical, at the protein level, to its counterparts in the seven other strains tested. This difference could lead to differential regulation of the site-specific recombinase of the IMESag-*rpsI* that allows a differential excision/circularization rate.

### 3.4. *In vivo* activity of the IMESag-*rpsI* conjugative transfer module

The conjugative transfer capacities of IMESag-*rpsI* from strain HRC were further investigated. Apart from a gene coding a putative relaxase (that we have designated *mobSag*), no other putative conjugative elements, such as coupling protein or elements participating in T4SS, were observed*.* The upstream and downstream regions of the *mobSag* gene encompassed a putative *oriT* and a type-A lagging-strand origin (*ssoA*) [43], respectively (figure 4*c*). The *oriT* of IMESag-*rpsI* (named *oriT*_Sag_) spans 42 bp and, similarly to the *oriT* of plasmid pMV158, contains three partially overlapping inverted repeats (IR1 to IR3). Based on its conservation among the *oriT* sequences of the pMV158 family [27], a possible nick site within the sequence 5′-GTGCG/T-3′ was also predicted (‘/’ denoting the phosphodiester bond cleaved by the cognate relaxase).

As IMESag-*rpsI* of strain HRC lacks any recognizable antibiotic resistance gene, exploration of its transfer abilities was tackled by cloning the mobilization module into a compatible and selectable plasmid. We chose pMV158 because it was isolated from *S. agalactiae*, harbours a tetracycline-resistance gene, and encodes the MobM relaxase [36,44]. We swapped the mobilization cassettes of pMV158 and IMESag-*rpsI* in two steps by: (i) construction of plasmid pMVS, which is a pMV158-derivative that lacks the mobilization cassette (*oriT*_pMV158_, *mobM*, and *ssoA*_pMV158_; figure 1), and (ii) cloning the mobilization module of IMESag-*rpsI* (*oriT*_Sag_, *mobSag*, and *ssoA*_Sag_) into pMVS. The resulting plasmid, pMVSag, has the replicative functions of pMV158 and the transfer functions of IMESag-*rpsI* (figure 1). Filter-mating assays were performed using an optimized approach and the frequency of conjugation was calculated as the number of transconjugants per recipient cell [38]. *Streptococcus pneumoniae* 708 cells carrying the auxiliary plasmid pAMβ1 and one of plasmids pMV158, pMVS or pMVSag were used as donors. As recipients, the isogenic novobiocin-resistant (Nov^R^) *S. pneumoniae* MP3008 strain was used (table 1) [3]. The mating experiments showed that pMVSag was efficiently transferred, albeit at a lower frequency than pMV158: 1.5 (±0.5) × 10^−5^ versus 22 (±2.1) × 10^−5^, respectively. No transconjugants were obtained when plasmid pMVS was used (below 10^−10^, the detection limit of the assay). These results demonstrate that the chromosomal region cloned into plasmid pMVSag contains all the necessary elements for its mobilization and, therefore, the IMESag-*rpsI* mobilization module is functional *in vivo*.

### 3.5. Characterization of the MobSag protein

A detailed *in silico* analysis revealed that the *mobSag* gene of strain HRC encodes a 418-amino acid protein, MobSag, which exhibits all the conserved motifs present in the N-terminal moiety of the MOB_V1_ family of conjugative relaxases [44]: (i) Motif I (HxxR; residues 22–25), of unknown function, (ii) Motif II (NYD/EL; residues 43–46), which contains the putative catalytic tyrosine of the MOB_V1_ relaxases [27]; and (iii) Motif III (HxDExxPHUH; residues 126–135), also known as the HUH motif (U being a non-polar residue), involved in coordination of a divalent metal (electronic supplementary material, figure S1*a*). The C-terminal domain of MobSag is predicted to be involved in the dimer formation, probably through a Leu-zipper (residues 346–367), as well as in the association of the protein to the cell membrane [45].

**Figure 6.**
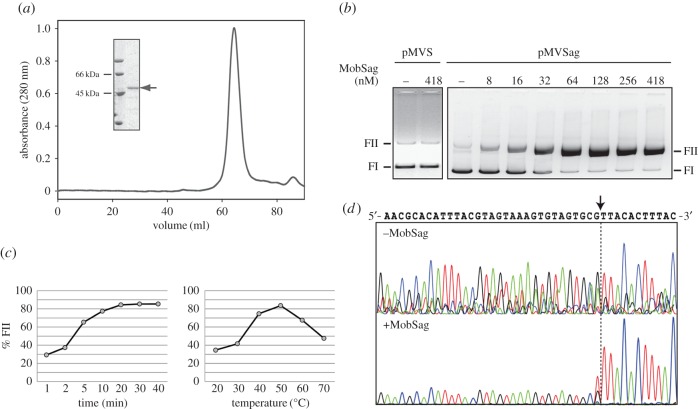
Functional characterization of the MobSag relaxase. (*a*) Elution profile of purified MobSag injected onto a HiLoad Superdex 200 gel filtration column (flow rate of 0.5 ml min^−1^ in buffer A. Inset shows the electrophoretic mobility of the main peak of the protein (arrow) on 12% SDS-PAGE; left, molecular size markers run on the same gel. (*b*) Relaxation of supercoiled DNA (8 nM) from plasmids pMVS or pMVSag after incubation (50°C, 20 min) without (−) or with purified MobSag protein (in buffer B containing 8 mM Mn^2+^). Relaxed (FII) and supercoiled (FI) plasmid forms are indicated. Protein concentrations were: 418 nM with pMVS (left panel), and 8, 16, 32, 64, 128, 256 and 418 nM with pMVSag (right panel). (*c*) Relaxation of pMVSag supercoiled DNA (8 nM) by MobSag relaxase (256 nM of protein in buffer B containing 8 mM Mn^2+^ at 50°C and different times (left panel) or at different temperatures for 20 min (right panel). Quantification of relaxed forms FII was done as indicated in Material and methods. (*d*) Determination of the MobSag nick site by means of Sanger DNA sequencing. Supercoiled DNA of pMVSag (8 nM) was incubated in absence (−) or presence (+) of MobSag as in panel (*b*). Relaxed FII DNA forms were purified and sequenced by using the primer nickSag-R (electronic supplementary material, table S1). Chromatograms of the coding strand are shown. A vertical arrow indicates the position of the nick site. The chromatograms of the non-coding strand did not show any significant variation of the peak intensity (not shown).

Overexpression and purification of the native MobSag protein was achieved following a previously described three-step protocol [36], rendering a final yield of 5 mg per litre of cell culture (purity above 98%). Determination of its N-terminal amino acid sequence showed that formylMet1 was removed, but the next 10 determined residues corresponded to the deduced amino acid sequence (SYVVARMAKY). Under denaturing conditions (SDS-PAGE), the protein migration agreed with the protein protomer's predicted size of 48.7 kDa (figure 6*a*). The analysis by gel filtration chromatography resulted in a single elution peak that fits with a MobSag homodimer (figure 6*a*). Prediction of the secondary structure of MobSag was achieved by computational and circular dichroism (CD) approaches (see electronic supplementary material, Methods). Employment of the program SABLE [46] showed the presence of the α/β-fold (α-helices alternating with β-strands), which is the typical distribution on the HUH-relaxases [15] (electronic supplementary material, figure S1*a*). This prediction was experimentally tested by CD in the far-UV region (electronic supplementary material, figure S1*b*). The CD spectrum of MobSag showed two minima at 208 nm and 222 nm, a typical feature of α-helical structures. Deconvolution of the CD spectra showed that the content of α-helices and β-strands agreed with the predicted secondary structure obtained by computational methods, and that the MobSag protein had a high α-helical content (electronic supplementary material, table S5). To determine the thermal stability of MobSag, CD spectroscopic analyses were performed at different temperatures (electronic supplementary material, figure S1*c*). At 40°C, there was a progressive loss of ellipticity indicating that denaturation of MobSag started around this temperature. Above 50°C, the ellipticity pronouncedly decreased as the temperature increased. Once the maximum temperature of 90°C was reached, the sample was cooled to the initial temperature (4°C), to study the reversibility of MobSag denaturation. The re-naturalized protein showed partial recovery of secondary structure, indicating that MobSag was partially refolded.

### 3.6. *In vitro* relaxase activity of MobSag

The relaxase activity of MobSag was demonstrated by *in vitro* nicking assays on plasmid supercoiled DNA. Different concentrations of purified protein were incubated with supercoiled DNA of pMVS (lacking *oriT*) or pMVSag. The results showed no DNA relaxed forms when plasmid pMVS was used as substrate, even at the highest protein concentration (figure 6*b*). However, in the *oriT*_Sag_-containing plasmid pMVSag, increasing the amount of MobSag led to a proportional generation of plasmid relaxed DNA forms; maximal relaxation (approx. 85%) was observed at a DNA : protein ratio of 1 : 6. Two other parameters relevant for the reaction were tested, namely incubation time and temperature (figure 6*c*). The optimal reaction conditions were found to be an incubation period of 20 min and a temperature of 50°C. Whether this latter finding is related to the favourable extrusion of the main hairpin at the *oriT*_Sag_ remains to be determined.

One important subject when addressing the interactions of a relaxase with its cognate *oriT* is the precise determination of the dinucleotide that it cleaves. This has been performed through tedious experiments that, in the case of MobM from pMV158, needed careful determination because the cleavage site by the relaxase (5′-GTGTG/TT-3′; ‘/’ being the cleavage site) needed different experimental approaches to disambiguate the nick site [47]. In the case of MobSag, the sequence predicted to contain the nick site was very similar: 5′-GTGcG/TT-3′ (figure 4*c*). To identify the nick site of relaxase MobSag, we developed a simple approach that could be extended to any relaxase protein. Purified MobSag protein (256 nM) was incubated with supercoiled DNA of pMVSag (8 nM) under optimal conditions (20 min, 50°C). The relaxed and uncleaved plasmid DNA forms were purified and subjected to Sanger DNA sequencing. The results clearly showed that the protein specifically cleaved at the 5′-GT-3′ phosphodiester bond in the loop of IR2 at the plasmid relaxase gene coding strand (figures 6*d* and 4). A detailed comparison of our present knowledge on the physicochemical and biochemical features of the two proteins MobM and MobSag is presented in table 3.

**Table 3.** Comparative features of relaxases MobM and MobSag.

	MobM	MobSag (this work)
*source*	plasmid pMV158 (Burdett, 1980)	IMESag-*rpsI* (*S. agalactiae* HRC chromosome)
*size*	494 residues (de Antonio *et al.*, 2004)	418 residues
*molecular weight* (*monomer*)	57,900 Dalton (MALDI-TOF) (Fernández-López *et al.*, 2013)	48 554 Dalton (predicted)
*N-terminal sequence*	SYMVARMQKM (Lorenzo-Díaz *et al.*, 2011)	SYVVARMAKY
*secondary structure*	60% α-helix, 4% β-strand, 36% random coils (de Antonio *et al.*, 2004)	55% α-helix, 10% β-strand, 31% turns, 15% unordered
*oligomeric state*	dimer (de Antonio *et al.*, 2004)	dimer
*frictional ratio (f/f*_*0*_)	1.65 (Fernández-López *et al.*, 2013)	1.34
*enzymatic activity*	DNA relaxase (Guzmán & Espinosa, 1997)	DNA relaxase
*nick site (“/”)*	5’-TAGTGTG/TTA-3’ (Guzmán & Espinosa, 1997)	5′-TAGTGCG/TTA-3′
*optimal relaxation temperature/time*	30°C (de Antonio *et al.*, 2004)/20 minutes (Guzmán & Espinosa, 1997)	50°C / 20 minutes
*in vivo mobilization activity*	yes (Farías & Espinosa, 2000; Lorenzo-Díaz & Espinosa, 2009; Smith *et al.*, 1980)	yes

## 4. Discussion

Mobilizable plasmids and IMEs may encode only a relaxase for their transfer and the rest of the conjugative machinery is provided either by an auxiliary plasmid or by chromosomally encoded elements like ICEs. This implies that the genetic information contained in plasmids and IMEs is kept at a minimum [11,27,48], according to the ‘travel light’ hypothesis for small genetic elements [49]. ICEs, IMEs and related transmissible elements are the most commonly found elements in *S. agalactiae*; they are a major driving force shaping the bacterial genome [6]. Although IMEs have been shown to be frequently found in bacteria [14], only a few IMEs have been experimentally characterized [50]. Here, we have performed a detailed analysis of the IMESag-*rpsI* within the chromosome of the GBS clinical isolate HRC and determined the nucleotide sequence of the bacterial isolate. IMESag-*rpsI* was previously identified in six human GBS isolates [7], and it harbours genes involved in recombination and conjugative transfer, as well as in replication and stable inheritance (figure 4) [11]. The phylogenetic study of 240 diverse strains allowed us to attain a general view of the IMESag-*rpsI* distribution and diversity in the GBS population (figure 2). Ten different IMESag-*rpsI* types were identified, types A and B representing the majority (79%) of them; these two types were heterogeneously distributed among four and five different CCs, respectively. In addition to single-nucleotide substitutions, the available data strongly support that recombination events have probably contributed to the observed diversity of IMESag-*rpsI*. Type-A IMESag-*rpsI* was found more frequently in CC1, whereas CC6, CC8, CC10 and CC19 were mainly populated by type-B IMESag-*rpsI*. The rest of the IMESag-*rpsI* types were poorly represented and mostly confined to a single CC (table 2). The conservation of IMESag-*rpsI* within CC1, CC6, CC8, CC10 and CC19 reflects its stability, probably due to the presence of a toxin–antitoxin gene cassette. Interestingly, search for this IME in 1143 streptococcal genomes showed it to be present in a single *S. dysgalactiae* subsp. *equisimilis* strain (WCHSDSE-1 contig_46). Therefore, despite its abundance in GBS, IMESag-*rpsI* seems to be restricted to this host and its origin remains unknown.

The presence of the same IMESag-*rpsI* type in strains belonging to different CCs suggests that this integrative element has been transmitted both vertically and horizontally, as has been shown for GBS strains harbouring Tn*5801* [51]. Although the mechanisms that induce ICE/IME excision from the chromosome are not fully understood, our results showed that the IMESag-*rpsI* from the HRC isolate was able to excise as circular intermediates when compared with other reference strains (figure 5). Excision of ICEs from the chromosome can be exceptional and dependent on growth conditions, as shown for ICE*Bs1* from *Bacillus subtilis* in which excision occurred only in 0.005% of the cell population [52]. It is worth pointing out that both strains 2603V/R and Spain-54 belong to CC19 and harbour a type-D IMESag-*rpsI*; thus it would be interesting to know whether the excision properties of IMESag-*rpsI* are type-dependent.

Excision of the IMESag-*rpsI* (figure 5) and the functionality of the conjugation module were demonstrated; the latter was achieved by cloning its mobilization module (*oriT*_Sag_ and *mobSag* gene) into a pMV158-derivative plasmid lacking mobilization functions (figure 1). Conjugation transfer of the element from the HRC strain is therefore highly probable. Furthermore, protein MobSag showed 85% similarity with the N-terminal region (the relaxase domain) of protein MobM (encoded by plasmid pMV158), which is the prototype of the MOB_V1_ family of conjugative relaxases [27]. Comparison of the MobSag amino acid sequences found in all IMESag-*rpsI* revealed a very high degree of conservation. Only six conserved amino acid changes, all polar uncharged residues, were identified (D93N, E105D, D341E, N370S, I394V and S396T). None of the substitutions were located within the three conserved domains of the N-terminal moiety [15], or in the Leu-zipper of the C-terminal [45]. *In vitro* relaxation assays showed that MobSag had optimal conditions that were comparable to those of MobM, except in temperature (50°C and 30°C, respectively) and in the amount of relaxed molecules (85% for MobSag and 60% for MobM; electronic supplementary material, table S5). As no other factors involved in conjugative transfer, such as a putative coupling protein or T4SS, were found within HRC-IMESag-*rpsI*, transfer of this element to a recipient cell would require the auxiliary machinery provided either by the chromosome or by an auxiliary plasmid.

Understanding the mobility of mobile genetic elements will help to unveil the complex organization of the *S. agalactiae* genomes. However, there are few studies devoted to analyse the prevalence, diversity and mobilization of IMEs. Only one recent work showing the ubiquity of an IME (integrated in the tRNALys CTT gene) and its role in the *S. agalactiae* genome plasticity has been reported so far [53]. Our results show that the high prevalence of IMESag-*rpsI* in GBS is a consequence of HGT, exchange of types and vertical transmission. A low fitness cost of the element in conjunction with the presence of a toxin–antitoxin gene module (probably involved in maintenance when the element is excised) probably contributes to the IMESag-*rpsI* stable inheritance. In conclusion, we have expanded knowledge of the mechanisms of bacterial evolution and acquisition of new genetic traits in GBS.

## Supplementary Material

RSOB-16-0084_Supplementary material_corrected

## Data accessibility

The data supporting this article have been uploaded as the electronic supplementary material.

## References

[1] Ochman H, Lawrence JG, Groisman EA 2000 Lateral gene transfer and the nature of bacterial innovation. Nature 405, 229–304. (doi:10.1038/35012500) 10.1038/35012500 10830951

[2] Wellington EM et al. 2013 The role of the natural environment in the emergence of antibiotic resistance in Gram-negative bacteria. Lancet Infect. Dis. 13, 155–165. (doi:10.1016/S1473-3099(12)70317-1) 23347633 10.1016/S1473-3099(12)70317-1

[3] Lorenzo-Díaz F, Espinosa M 2009 Lagging strand DNA replication origins are required for conjugal transfer of the promiscuous plasmid pMV158. J. Bacteriol. 191, 720–727. (doi:10.1128/JB.01257-08) 19028894 10.1128/JB.01257-08 PMC2632109

[4] Edmond KM, Kortsalioudaki C, Scott S, Schrag SJ, Zaidi AKM, Cousens S, Heath PT 2012 Group B streptococcal disease in infants aged younger than 3 months: systematic review and meta-analysis. The Lancet 379, 547–556. (doi:10.1016/S0140-6736(11)61651-6) 10.1016/S0140-6736(11)61651-6 22226047

[5] Tettelin H et al. 2005 Genome analysis of multiple pathogenic isolates of *Streptococcus agalactiae*: implications for the microbial ‘pan-genome’. Proc. Natl Acad. Sci. USA 102, 13 950–13 955. (doi:10.1073/pnas.0506758102) 10.1073/pnas.0506758102 PMC1216834 16172379

[6] Brochet M, Rusniok C, Couve E, Dramsi S, Poyart C, Trieu-Cuot P, Kunst F, Glaser P 2008 Shaping a bacterial genome by large chromosomal replacements, the evolutionary history of *Streptococcus agalactiae*. Proc. Natl Acad. Sci. USA 105, 15 961–15 966. (doi:10.1073/pnas.0803654105) 10.1073/pnas.0803654105 PMC2572952 18832470

[7] Brochet M, Couve E, Glaser P, Guedon G, Payot S 2008 Integrative conjugative elements and related elements are major contributors to the genome diversity of *Streptococcus agalactiae*. J. Bacteriol. 190, 6913–6917. (doi:10.1128/JB.00824-08) 18708498 10.1128/JB.00824-08 PMC2566197

[8] Burrus V, Pavlovic G, Decaris B, Guédon G 2002 The ICE*St*1 element of *Streptococcus thermophilus* belongs to a large family of integrative and conjugative elements that exchange modules and change their specificity of integration. Plasmid 48, 77–97. (doi:10.1016/S0147-619X(02)00102-6) 12383726 10.1016/s0147-619x(02)00102-6

[9] Burrus V, Pavlovic G, Decaris B, Guédon G 2002 Conjugative transposons: the tip of the iceberg. Mol. Microbiol. 46, 601–610. (doi:10.1046/j.1365-2958.2002.03191.x) 12410819 10.1046/j.1365-2958.2002.03191.x

[10] Burrus V, Waldor MK 2004 Shaping bacterial genomes with integrative and conjugative elements. Res. Microbiol. 155, 376–386. (doi:10.1016/j.resmic.2004.01.012) 15207870 10.1016/j.resmic.2004.01.012

[11] Bellanger X, Payot S, Leblond-Bourget N, Guédon G 2014 Conjugative and mobilizable genomic islands in bacteria: evolution and diversity. FEMS Microbiol. Rev. 38, 720–760. (doi:10.1111/1574-6976.12058) 24372381 10.1111/1574-6976.12058

[12] Treangen TJ, Rocha EPC 2011 Horizontal transfer, not duplication, drives the expansion of protein families in prokaryotes. PLoS Genet. 7, e1001284 (doi:10.1371/journal.pgen.1001284) 21298028 10.1371/journal.pgen.1001284 PMC3029252

[13] Waldor MK 2010 Mobilizable genomic islands: going mobile with *oriT* mimicry. Mol. Microbiol. 78, 537–540. (doi:10.1111/j.1365-2958.2010.07365.x) 21038479 10.1111/j.1365-2958.2010.07365.x PMC3058610

[14] Guglielmini J, Quintais L, Garcillan-Barcia MP, de la Cruz F, Rocha EPC 2011 The repertoire of ICE in prokaryotes underscores the unity, diversity, and ubiquity of conjugation. PLoS Genet. 7, e1002222 (doi:10.1371/journal.pgen.1002222) 21876676 10.1371/journal.pgen.1002222 PMC3158045

[15] Chandler M, de la Cruz F, Dyda F, Hickman AB, Moncalian G, Ton-Hoang B 2013 Breaking and joining single-stranded DNA: the HUH endonuclease superfamily. Nat. Rev. Microbiol. 11, 525–538. (doi:10.1038/nrmicro3067) 23832240 10.1038/nrmicro3067 PMC6493337

[16] Glaser P et al. 2002 Genome sequence of *Streptococcus agalactiae*, a pathogen causing invasive neonatal disease. Mol. Microbiol. 45, 1499–1513. (doi:10.1046/j.1365-2958.2002.03126.x) 12354221 10.1046/j.1365-2958.2002.03126.x

[17] Horodniceanu T, Bougueleret L, El-Solh N, Bouanchaud DH, Chabbert YA 1979 Conjugative R plasmids in *Streptococcus agalactiae* (group B). Plasmid 2, 197–206. (doi:10.1016/0147-619X(79)90038-6) 109871 10.1016/0147-619x(79)90038-6

[18] Da Cunha V et al. 2014 *Streptococcus agalactiae* clones infecting humans were selected and fixed through the extensive use of tetracycline. Nat. Commun. 5, 4544 (doi:10.1038/ncomms5544) 25088811 10.1038/ncomms5544 PMC4538795

[19] Tettelin H et al. 2002 Complete genome sequence and comparative genomic analysis of an emerging human pathogen, serotype V *Streptococcus agalactiae*. Proc. Natl Acad. Sci. USA 99, 12 391–12 396. (doi:10.1073/pnas.182380799) 10.1073/pnas.182380799 PMC129455 12200547

[20] Lacks S, Greenberg B 1977 Complementary specificity of restriction endonucleases of *Diplococcus pneumoniae* with respect to DNA methylation. J. Mol. Biol. 114, 153–168. (doi:10.1016/0022-2836(77)90289-3) 20509 10.1016/0022-2836(77)90289-3

[21] Studier FW, Moffatt BA 1986 Use of bacteriophage T7 RNA polymerase to direct selective high-level expression of cloned genes. J. Mol. Biol. 189, 113–130. (doi:10.1016/0022-2836(86)90385-2) 3537305 10.1016/0022-2836(86)90385-2

[22] Burdett V 1980 Identification of tetracycline-resistant R-plasmids in *Streptococcus agalactiae* (group B). Antimicrob. Agents Chemother. 18, 753–760. (doi:10.1128/AAC.18.5.753) 7004347 10.1128/aac.18.5.753 PMC284087

[23] Janniere L, Bruand C, Ehrlich SD 1990 Structurally stable *Bacillus subtilis* cloning vectors. Gene 87, 53–61. (doi:10.1016/0378-1119(90)90495-D) 2110098 10.1016/0378-1119(90)90495-d

[24] Lacks S 1966 Integration efficiency and genetic recombination in pneumococcal transformation. Genetics 53, 207–235. 4379022 10.1093/genetics/53.1.207 PMC1211003

[25] Ruiz-Cruz S, Solano-Collado V, Espinosa M, Bravo A 2010 Novel plasmid-based genetic tools for the study of promoters and terminators in *Streptococcus pneumoniae* and *Enterococcus faecalis*. J. Microb. Meth. 83, 156–163. (doi:10.1016/j.mimet.2010.08.004) 10.1016/j.mimet.2010.08.004 20801171

[26] del Solar G, Díaz R, Espinosa M 1987 Replication of the streptococcal plasmid pMV158 and derivatives in cell-free extracts of *Escherichia coli*. Mol. Gen. Genet. 206, 428–435. (doi:10.1007/BF00428882) 3035343 10.1007/BF00428882

[27] Lorenzo-Díaz F, Fernández-López C, Garcillán-Barcia MP, Espinosa M 2014 Bringing them together: plasmid pMV158 rolling circle replication and conjugation under an evolutionary perspective. Plasmid 74, 15–31. (doi:10.1016/j.plasmid.2014.05.004) 24942190 10.1016/j.plasmid.2014.05.004 PMC7103276

[28] Treangen TJ, Ondov BD, Koren S, Phillippy AM 2014 The Harvest suite for rapid core-genome alignment and visualization of thousands of intraspecific microbial genomes. Genome Biol. 15, 524 (doi:10.1186/s13059-014-0524-x) 25410596 10.1186/s13059-014-0524-x PMC4262987

[29] Bruen TC, Philippe H, Bryant D 2006 A simple and robust statistical test for detecting the presence of recombination. Genetics 172, 2665–2681. (doi:10.1534/genetics.105.048975) 16489234 10.1534/genetics.105.048975 PMC1456386

[30] Price MN, Dehal PS, Arkin AP 2010 FastTree 2–approximately maximum-likelihood trees for large alignments. PLoS ONE 5, e9490 (doi:10.1371/journal.pone.0009490) 20224823 10.1371/journal.pone.0009490 PMC2835736

[31] Letunic I, Bork P 2011 Interactive Tree Of Life v2: online annotation and display of phylogenetic trees made easy. Nucleic Acids Res. 39, W475–W478. (doi:10.1093/nar/gkr201) 21470960 10.1093/nar/gkr201 PMC3125724

[32] Sullivan MJ, Petty NK, Beatson SA 2011 Easyfig: a genome comparison visualizer. Bioinformatics 27, 1009–1010. (doi:10.1093/bioinformatics/btr039) 21278367 10.1093/bioinformatics/btr039 PMC3065679

[33] Kosakovsky Pond SL, Posada D, Gravenor MB, Woelk CH, Frost SDW 2006 GARD: a genetic algorithm for recombination detection. Bioinformatics 22, 3096–3098. (doi:10.1093/bioinformatics/btl474) 17110367 10.1093/bioinformatics/btl474

[34] Espinosa M, López P, Pérez-Ureña MT, Lacks SA 1982 Interspecific plasmid transfer between *Streptococcus pneumoniae* and *Bacillus subtilis*. Mol. Gen. Genet. 188, 195–201. (doi:10.1007/BF00332675) 6296628 10.1007/BF00332675

[35] Dower WJ, Miller JF, Ragsdale CW 1988 High efficiency transformation of *E. coli* by high voltage electroporation. Nucleic Acids Res. 16, 6127–6145. (doi:10.1093/nar/16.13.6127) 3041370 10.1093/nar/16.13.6127 PMC336852

[36] Lorenzo-Díaz F, Dostál L, Coll M, Schildbach JF, Menendez M, Espinosa M 2011 The MobM-relaxase domain of plasmid pMV158: thermal stability and activity upon Mn^2+^- and DNA specific-binding. Nucleic Acids Res. 39, 4315–4329. (doi:10.1093/nar/gkr049) 21296755 10.1093/nar/gkr049 PMC3105389

[37] Fernández-López C, Lorenzo-Díaz F, Pérez-Luque R, Rodríguez-González L, Boer R, Lurz R, Bravo A, Coll M, Espinosa M 2013 Nicking activity of the pMV158 MobM relaxase on cognate and heterologous origins of transfer. Plasmid 70, 120–130. (doi:10.1016/j.plasmid.2013.03.004) 23562993 10.1016/j.plasmid.2013.03.004

[38] Lorenzo-Díaz F, Espinosa M 2009 Large-scale filter mating assay for intra- and inter-specific conjugal transfer of the promiscuous plasmid pMV158 in Gram-positive bacteria. Plasmid 61, 65–70. (doi:10.1016/j.plasmid.2008.09.005) 18848959 10.1016/j.plasmid.2008.09.005

[39] Baez-Ortega A, Lorenzo-Diaz F, Hernandez M, Gonzalez-Vila CI, Roda-Garcia JL, Colebrook M, Flores C 2015 IonGAP: integrative bacterial genome analysis for Ion Torrent sequence data. Bioinformatics 31, 2870–2873. (doi:10.1093/bioinformatics/btv283) 25953799 10.1093/bioinformatics/btv283

[40] Zbinden A, Mueller NJ, Tarr PE, Spröer C, Keller PM, Bloemberg GV 2012 *Streptococcus tigurinus* sp. nov., isolated from blood of patients with endocarditis, meningitis and spondylodiscitis. Int. J. Syst. Evol. Microbiol. 62, 2941–2945. (doi:10.1099/ijs.0.038299-0) 22357776 10.1099/ijs.0.038299-0

[41] Zbinden A, Quiblier C, Hernandez D, Herzog K, Bodler P, Senn MM, Gizard Y, Schrenzel J, François P 2014 Characterization of *Streptococcus tigurinus* small-colony variants causing prosthetic joint infection by comparative whole-genome analyses. J. Clin. Microbiol. 52, 467–474. (doi:10.1128/JCM.02801-13) 24478475 10.1128/JCM.02801-13 PMC3911336

[42] Giovanetti E, Brenciani A, Tiberi E, Bacciaglia A, Varaldo PE 2012 ICES*p2905*, the *erm*(TR)-*tet*(O) element of *Streptococcus pyogenes*, is formed by two independent integrative and conjugative elements. Antimicrob. Agents Chemother. 56, 591–594. (doi:10.1128/AAC.05352-11) 21986826 10.1128/AAC.05352-11 PMC3256084

[43] Kramer MG, Espinosa M, Misra TK, Khan SA 1998 Lagging strand replication of rolling-circle plasmids: specific recognition of the *ssoA*-type origins in different Gram-positive bacteria. Proc. Natl Acad. Sci. USA 95, 10 505–10 510. (doi:10.1073/pnas.95.18.10505) 10.1073/pnas.95.18.10505 PMC27924 9724733

[44] Garcillan-Barcia MP, Francia M, de la Cruz F 2009 The diversity of conjugative relaxases and its application in plasmid classification. FEMS Microbiol. Rev. 33, 657–687. (doi:10.1111/j.1574-6976.2009.00168.x) 19396961 10.1111/j.1574-6976.2009.00168.x

[45] de Antonio C, Farias ME, de Lacoba MG, Espinosa M 2004 Features of the plasmid pMV158-encoded MobM, a protein involved in its mobilization. J. Mol. Biol. 335, 733–743. (doi:10.1016/j.jmb.2003.11.017) 14687570 10.1016/j.jmb.2003.11.017

[46] Adamczak R, Porollo A, Meller J 2005 Combining prediction of secondary structure and solvent accessibility in proteins. Proteins 59, 467–475. (doi:10.1002/prot.20441) 15768403 10.1002/prot.20441

[47] Guzmán LM, Espinosa M 1997 The mobilization protein, MobM, of the streptococcal plasmid pMV158 specifically cleaves supercoiled DNA at the plasmid *oriT*. J. Mol. Biol. 266, 688–702. (doi:10.1006/jmbi.1996.0824) 9102462 10.1006/jmbi.1996.0824

[48] Fernández-López C, Bravo A, Ruiz-Cruz S, Solano-Collado V, Garsin DA, Lorenzo-Díaz F, Espinosa M 2014 Mobilizable rolling-circle replicating plasmids from Gram-positive bacteria: a low-cost conjugative transfer. Microbiol. Spectr. 2, 8 (doi:10.1128/microbiolspec.PLAS-0008-2013) 25606350 10.1128/microbiolspec.PLAS-0008-2013 PMC4297665

[49] Espinosa M, del Solar G, Rojo F, Alonso JC 1995 Plasmid rolling circle replication and its control. FEMS Microbiol. Lett. 130, 111–120. (doi:10.1111/j.1574-6968.1995.tb07707.x) 7649431 10.1111/j.1574-6968.1995.tb07707.x

[50] Shoemaker N, Wang G, Salyers A 2000 Multiple gene products and sequences required for excision of the mobilizable integrated *Bacteroides* element NBU1. J. Bacteriol. 182, 928–936. (doi:10.1128/JB.182.4.928-936.2000) 10648516 10.1128/jb.182.4.928-936.2000 PMC94366

[51] León-Sampedro R, Novais C, Peixe L, Baquero F, Coque TM 2016 Diversity and evolution of the Tn*5801*-*tet*(M)-like integrative–conjugative elements among *Enterococcus*, *Streptococcus*, and *Staphylococcus*. Antimicrob. Agents Chemother. 60, 1736–1746. (doi:10.1128/AAC.01864-15) 26729505 10.1128/AAC.01864-15 PMC4775984

[52] Lee CA, Auchtung JM, Monson RE, Grossman AD 2007 Identification and characterization of *int* (integrase), *xis* (excisionase) and chromosomal attachment sites of the integrative and conjugative element ICE*Bs*1 of *Bacillus subtilis*. Mol. Microbiol. 66, 1356–1369. (doi:10.1111/j.1365-2958.2007.06000.x) 18005101 10.1111/j.1365-2958.2007.06000.x

[53] Puymège A, Bertin S, Guédon G, Payot S 2015 Analysis of *Streptococcus agalactiae* pan-genome for prevalence, diversity and functionality of integrative and conjugative or mobilizable elements integrated in the tRNALys CTT gene. Mol. Genet. Genomics 290, 1727–1740. (doi:10.1007/s00438-015-1031-9) 25832353 10.1007/s00438-015-1031-9

