# Supplementary material for: Streptococcal group B integrative and mobilizable element IMESag-rpsI encodes a functional relaxase involved in its transfer
Source: Open Biol. 2016 Oct 5;6(10):160084. doi: 10.1098/rsob.160084 (PMC5090054; doi:10.1098/rsob.160084)
Supplement: RSOB-16-0084_Supplementary material_corrected [file rsob160084supp1.pdf]

Supplementary Material

Streptococcal Group B Integrative and Mobilizable Element IMESag-*rpsI* Encodes a Functional Relaxase Involved in its Transfer

Fabian Lorenzo-Diaz, Cris Fernandez-Lopez, Pierre-Emmanuel Douarre, Adrian Baez-Ortega, Carlos Flores, Philippe Glaser and Manuel Espinosa

Methods

Secondary structure predictions

Secondary-structure predictions of MobSag were performed with programs SABLE (Adamczak *et al.*, 2005), JPred (Cole *et al.*, 2008), PredictProtein (Rost *et al.*, 2004), and PSIPred (McGuffin & D.T., 2000).

Circular dichroism (CD) and thermal stability assays

CD spectra of MobSag protein samples (20 µM in buffer CD, containing 10 mM potassium phosphate and 100 mM ammonium sulphate, pH 7.6) were acquired in a Jasco J-810 spectropolarimeter employing 0.2-mm path length quartz cuvettes. Spectra were recorded over a wavelength range from 185 to 260 nm (far-UV) at 4ºC, at a scan speed of 50 nm/min. The results were expressed as mean residue ellipticity [Θ] at a given wavelength. To obtain structural information, the CD data were deconvoluted using SELCON3 (Sreerama & Woody, 2000), CONTINLL (Provencher & Glöckner, 1981), CDSSTR (Compton & Johnson, 1986), and K2D (Andrade *et al.*, 1993) algorithms, available at the DichroWeb site (Whitmore & Wallace, 2004). Temperature-induced changes in the MobSag protein secondary structure were measured at wavelengths ranging from 202 to 260 nm by increasing the temperature from 4ºC to 90ºC at 50ºC/h. Finally, the samples were cooled to the initial temperature (4ºC) and a spectrum at the same wavelength range was recorded. Changes in ellipticity were recorded at 220 nm in a 1-mm optical path length quartz cuvette. Data acquirement and processing were carried out using the Jasco Spectra-Manager software and CD spectra at different temperatures were represented with SigmaPlot v10.0.

Supplementary Table S1. Oligonucleotides used in this study.

Name	Sequence (5´ to 3´)[a]	Used for
INV-F	**AGGCCT**ACTCTATTCGCCTTTTGACTTTT	Constructing plasmid pMVS
INV-R	CCGACCAAAACCATAAAACC	
Sag1-F	TGGCAATTACC**GCTAGC**ATCTCGGCGA	Cloning *mobSag* gene
Sag1-R	CCTGACGA**CTCGAG**CCCATCTAGT	
Sag2-F	AGCGAAGAGCTGTCTGCAAG	Cloning mobilization module ($oriT_{Sag}$, *mobSag*, $ssoA_{Sag}$)
Sag2-R	TCCTTACTCATTTATTCCATGGTTC	
cIME-F	GCTTGGAATGTGACAGCTC	Detecting circular forms of IMESag-*rpsI*
cIME-R	GCAATTTGCGGACTATCTC	
nickSag-R	TAGCCGTCAACTGACCTGACT	Identifying MobSag nick site

[a]Oligonucleotides Sag1-F, Sag1-R and INV-F include recognition sites (highlighted in bold letters) for *Nhe*I, *Ava*I and *Stu*I enzymes, respectively. The base changes introduced to produce the recognition sites are underlined.

Supplementary Table S2. Divergence matrix among the different IMESag-*rpsI* types.

IMESag-*rpsI*			SNPs / kb[b]									
**Type**	**Strain (acc. number)[a]**	**Length (bp)**	**A**	**B**	**C**	**D**	**E**	**F**	**G**	**H**	**I**	**J**
A	HRC (SRS985592)	9,153	0.7									
B	RBH11 (ERS046925)	9,221	47.1	0.7								
C	CZ-NI-004 (ERS039685)	9,357	23.4	57.8	0.0							
D	Spain-54 (ERS337449)	9,383	15.1	45.9	42.4	0.1						
E	CRBIP22.117 (ERS337446)	9,130	48.3	15.7	63.3	34.8	0.0					
F	CZ-NI-007 (ERS039688)	9,220	43.1	5.3	55.3	43.4	19.3	0.5				
G	CRBIP22.113 (ERS337515)	9,498	33.6	38.6	46.9	28.3	29.9	40.8	0.0			
H	Madagascar-IP-7 (ERS337519)	9,531	15.4	41.7	46.9	27.1	42.4	37.8	46.6	0.0		
I	B42VD (ERS046909)	9,127	51.4	45.4	46.6	39.3	31.6	47.7	16.5	49.0	0.0	
J	CCH208800621 (ERS337459)	9,511	33.8	28.5	54.6	43.7	30.4	24.6	40.3	33.4	45.4	0.0

[a]The name of the reference strains employed for diversity estimations are annotated and their accession numbers given in parenthesis (Database: http://sra.dnanexus.com)
[b]Single nucleotide polymorphism (SNPs) per kilobase (kb).

Supplementary Table S3. GBS strains used for HRC comparative genome analysis**.**

Clonal Complex (CC)	Strain[a]	Source	NCBI nucleotide	Size (Mb)	GC%	Genes	Proteins	Sequence Type	SNPs to HRC[b]
CC1	SS1	Human	NZ_CP010867.1	2.1	35.5	2,106	1,983	ST1	78
CC1	09mas018883	Bovine	NC_021485.1	2.1	35.5	2,153	2,036	ST1	110
CC1	NGBS061	Human	NZ_CP007631.1	2.2	35.5	2,249	2,118	ST459	1,936
CC6-8-10	GD201008-001	Fish	NC_018646.1	2.0	35.6	2,061	1,939	ST7	3,754
CC6-8-10	A909	Human	NC_007432.1	2.0	35.6	2,154	2,032	ST7	3,787
CC19	2603V/R	Human	NC_004116.1	2.0	35.6	2,279	2,127	ST110	4,064
CC23	NEM316	Human	NC_004368.1	2.0	35.6	2,217	2,106	ST23	5,168
CC22	GBS2-NM	Human	NZ_CP007571.1	2.0	35.9	2,202	2,069	ST22	6,033
CC22	GBS6	Human	NZ_CP007572.1	2.0	35.8	2,223	2,092	ST22	6,054
CC22	GBS1-NY	Human	NZ_CP007570.1	2.0	35.9	2,227	2,099	ST22	6,059
CC26	CNCTC 10/84	Human	NZ_CP006910.1	2.0	35.4	2,035	1,908	ST26	6,189
CC17	COH1	Human	NZ_HG939456.1	2.0	35.4	2,069	1,932	ST17	6,241
CC23	NGBS572	Human	NZ_CP007632.1	2.0	35.5	2,069	1,945	ST452	6,283
Other CC	ILRI112	Dromedary	HF952106.1	2.0	35.3	2,173	2,073	ST617	6,902
Other CC	ILRI005	Dromedary	NC_021486.1	2.0	35.4	2,180	2,025	ST609	7,283
CC552	2-22	Fish	NC_021195.1	2.0	35.5	1,865	1,650	ST261	7,722
CC552	SA20-06	Fish	NC_019048.1	2.0	35.6	1,863	1,668	ST552	7,730

[a]Available complete genome sequences (as of 2015-08-21): www.ncbi.nlm.nih.gov/genome/genomes/186#
[b]Single nucleotide polymorphism (SNPs) calculated with IonGAP platform (http://iongap.hpc.iter.es/iongap). Genotype confidence greater or equal to 10.

Supplementary Table S4. Putative mobile genetic elements found in the genome of strain HRC.

Element	Inserted in gene (position, strand)	Size (kb)	No. predicted genes	BLASTn best hit		
				Genome (GenBank accession)	Coordinate (5' end)	Identity (%)
Prophage	*comCG* (CDS, plus)	37	55	*S. agalactiae* SS1 (CP010867.1)	188,983	99
IME-*rpsI*	*rpsI* (3', plus)	22	26	*S. agalactiae* SS1 (CP010867.1)	230,695	99
CIME-*tRNAThr*	*tRNA*-Thr (3', minus)	3	6	*S. agalactiae* SS1 (CP010867.1)	461,664	100
ICE-*rumA*	*rumA* (3', plus)	54	46	*S. pyogenes* ICE*Sp2905* (FR691055.1)	1,659	66
ICE-Tn*916*[a]	*rib* (3', minus)	18	22	*S. agalactiae* SS1 (CP010867.1)	663,576	99
CIME-*guaA*	*guaA* (3', minus)	10	11	*S. agalactiae* SS1 (CP010867.1)	996,205	99
CIME-*rplL*	*rplL* (3', minus)	10	11	*S. agalactiae* SS1 (CP010867.1)	1,293,211	99
CIME-*tRNALys*	*tRNA*-Lys (3', plus)	7	13	*S. agalactiae* 09mas018883 (NC_021485.1)	1,916,558	99
CIME-*rpmG*	*rpmG* (3', plus)	8	11	*S. agalactiae* SS1 (CP010867.1)	2,008,224	99
Plasmid pST2426	N/A (extrachromosomal)	2.5	3	*S. tigurinus* 2426 plasmid pST2426 (NZ_ASXA01000016.1)	N/A	100

[a]Sublineage Tn*916*-1, according to (Da Cunha *et al.*, 2014).

N/A, not applicable.

Supplementary Table S5. Secondary structure content of MobSag.

Prediction program	α-helix	β-strand	Turns	Unordered
SABLE	66.9	7.0	N.D.	26.1
JPred3	66.9	8.6	N.D.	24.5
PSIPred	66.2	5.8	N.D.	28.0
PredictProtein	73.9	7.2	N.D.	N.D.
Average ± S.D.	68.5±3.6	7.2±1.1	N.D.	26.2±1.8
**Deconvolution method**	**α-helix**	**β-strand**	**Turns**	**Unordered**
SELCON3	42.7	15.3	46.6	25.5
CONTIN	53.8	7.9	33.4	4.9
CDSSTR	62.0	10.0	13.0	15.0
K2D	63.0	6.0	N.D.	N.D.
Average ± S.D.	55.4±9.4	9.8±4.0	31.0±16.9	15.1±10.3

Data and standard deviations are expressed in percentages. S.D.: Standard deviation; N.D.: Not determined.

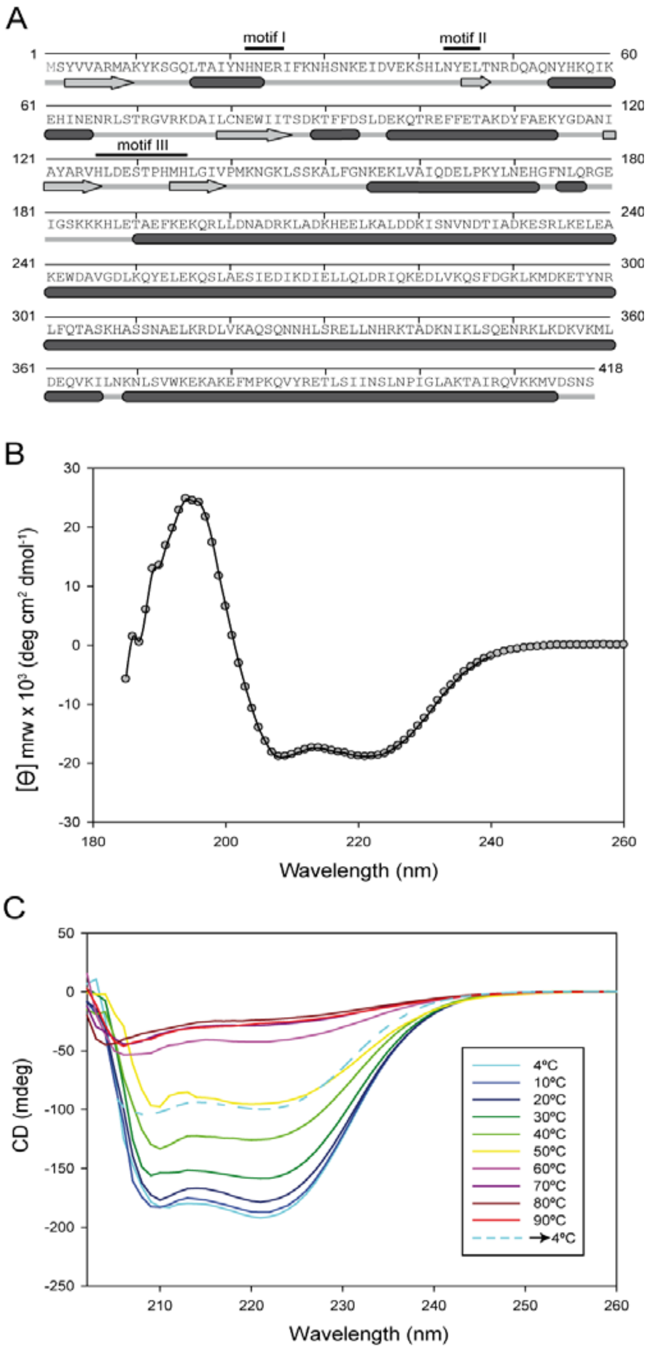

Supplementary Figure S1. Secondary structure and thermal stability of protein MobSag. **A)** *In silico* prediction of the secondary structure of MobSag obtained from the SABLE program (Adamczak *et al*., 2005). The predicted secondary structure (α-helices illustrated as dark grey cylinders and β-sheets as light grey arrows) are depicted beneath the MobSag amino acid sequence. Conserved N-terminal motifs of the MOB_{V1} family relaxases are indicated: motif I, HxxR (unknown function); motif II, NYEL (harbouring the proposed catalytic residue); motif III, HxDExxPHUH (involved in metal ion coordination). **B)** Circular Dichroism (CD) spectrum of MobSag (20 μM) in the far-UV region (185-260 nm) at 4ºC. The spectrum was obtained by subtracting the buffer spectrum data, measured under identical conditions. Experimental data (grey circles) and the fitted data using the CDSSTR method (continuous line) are shown. **C)** Temperature-associated changes in the secondary structure of MobSag. Far-UV CD spectra of the protein (20 μM) were recorded during thermal denaturing (solid lines) and refolding (heated sample cooled down to 4ºC; dotted line) processes.

References

Adamczak, R., A. Porollo & J. Meller, (2005) Combining prediction of secondary structure and solvent accessibility in proteins. *Proteins* **59**: 467-475.

Andrade, M.A., P. Chacón, J.J. Merolo & F. Morán, (1993) Evaluation of secondary structure of proteins from UV circular dichroism spectra using an unsupervised learning neural network. *Protein Eng.* **6**: 443-454.

Burdett, V., (1980) Identification of tetracycline-resistant R-plasmids in *Streptococcus agalactiae* (group B). *Antimicrob. Agents Chemother.* **18**: 753 - 760.

Cole, C., J.D. Barber & G.J. Barton, (2008) The Jpred 3 secondary structure prediction server. *Nucl. Acids Res.* **36**: 197-201.

Compton, L.A. & W.C. Johnson, Jr., (1986) Analysis of protein circular dichroism spectra for secondary structure using a simple matrix multiplication. *Anal. Biochem.* **155**: 155-167.

Da Cunha, V., M.R. Davies, P.E. Douarre, I. Rosinski-Chupin, I. Margarit, S. Spinali, T. Perkins, P. Lechat, N. Dmytruk, E. Sauvage, L. Ma, B. Romi, M. Tichit, M.J. Lopez-Sanchez, S. Descorps-Declere, E. Souche, C. Buchrieser, P. Trieu-Cuot, I. Moszer, D. Clermont, D. Maione, C. Bouchier, D.J. McMillan, J. Parkhill, J.L. Telford, G. Dougan, M.J. Walker, D. Consortium, M.T. Holden, C. Poyart & P. Glaser, (2014) *Streptococcus agalactiae* clones infecting humans were selected and fixed through the extensive use of tetracycline. *Nat. Commun.* **5**: 4544.

de Antonio, C., M.E. Farias, M.G. de Lacoba & M. Espinosa, (2004) Features of the plasmid pMV158-encoded MobM, a protein involved in its mobilization. *J. Mol. Biol.* **335**: 733-743.

Farías, M.E. & M. Espinosa, (2000) Conjugal transfer of plasmid pMV158: uncoupling of the pMV158 origin of transfer from the mobilization gene *mobM*, and modulation of pMV158 transfer in *Escherichia coli* mediated by IncP plasmids. *Microbiology* **146**: 2259-2265.

Fernández-López, C., F. Lorenzo-Díaz, R. Pérez-Luque, L. Rodríguez-González, R. Boer, R. Lurz, A. Bravo, M. Coll & M. Espinosa, (2013) Nicking activity of the pMV158 MobM relaxase on cognate and heterologous origins of transfer. *Plasmid* **70**: 120-130.

Guzmán, L.M. & M. Espinosa, (1997) The mobilization protein, MobM, of the streptococcal plasmid pMV158 specifically cleaves supercoiled DNA at the plasmid *oriT*. *J. Mol. Biol.* **266**: 688-702.

Lorenzo-Díaz, F., L. Dostál, M. Coll, J.F. Schildbach, M. Menendez & M. Espinosa, (2011) The MobM-relaxase domain of plasmid pMV158: thermal stability and activity upon Mn^{2+}-and DNA specific-binding. *Nucl. Acids Res.* **39**: 4315-4329.

Lorenzo-Díaz, F. & M. Espinosa, (2009) Lagging strand DNA replication origins are required for conjugal transfer of the promiscuous plasmid pMV158. *J. Bacteriol.* **191**: 720–727.

McGuffin, B.K. & J. D.T., (2000) The PSIPRED protein structure prediction server. *Bioinformatics* **16**: 404-405.

Provencher, S.W. & J. Glöckner, (1981) Estimation of globular protein secondary structure from circular dichroism. *Biochemistry* **20**: 33-37.

Rost, B., G. Yachdav & J. Liu, (2004) The PredictProtein server. *Nucl. Acids Res.* **32**: W321-326.

Smith, M.D., N.B. Shoemaker, V. Burdett & W.R. Guild, (1980) Transfer of plasmids by conjugation in *Streptococcus pneumoniae*. *Plasmid* **3**: 70-79.

Sreerama, N. & R.W. Woody, (2000) Estimation of Protein Secondary Structure from Circular Dichroism Spectra: Comparison of CONTIN, SELCON, and CDSSTR Methods with an Expanded Reference Set. *Anal. Biochem.* **287**: 252-260.

Whitmore, L. & B.A. Wallace, (2004) DICHROWEB, an online server for protein secondary structure analyses from circular dichroism spectroscopic data. *Nucl. Acids Res.* **32**: 668-673 (Web server issue).
